# Miliary Tuberculosis Induced Acute Liver Failure

**DOI:** 10.1155/2015/759341

**Published:** 2015-09-07

**Authors:** Tayfur Toptas, Birkan Ilhan, Huseyin Bilgin, Elif Dincses, Osman Ozdogan, Isik Kaygusuz-Atagunduz, Zekaver Odabasi, Volkan Korten, Tulin Firatli-Tuglular

**Affiliations:** ^1^Department of Hematology, Van Training and Research Hospital, 65300 Van, Turkey; ^2^Department of Internal Medicine, Marmara University Hospital, Istanbul, Turkey; ^3^Department of Infectious Diseases, Marmara University Hospital, Istanbul, Turkey; ^4^Department of Gastroenterology, Marmara University Hospital, Istanbul, Turkey; ^5^Department of Hematology, Marmara University Hospital, Istanbul, Turkey

## Abstract

Hepatobiliary tuberculosis is uncommon even in endemic countries. It is associated with a high mortality and is even diagnosed early in the disease course. Acute liver failure (ALF) caused by tuberculosis bacilli has been reported in only a few reports. All previous cases have been diagnosed by postmortem examination. Time to antituberculosis treatment is very critical. In case of suggestive findings on clinical and radiologic examination, antituberculosis treatment should be initiated immediately. Drug use can be a challenge in patients with ALF. However, as long as the other possible causes of ALF can be excluded and hepatotoxic drugs were avoided during the early course of treatment, such a highly fatal presentation of tuberculosis can be treated safely. Here, we report a case of acute liver failure as a presentation of miliary tuberculosis. He was treated successfully with antituberculosis treatment.

## 1. Introduction

Miliary TB is a common disease in TB-endemic countries. It is characterized by the appearance of small (<2 mm) nodules in lung parenchyma.* Mycobacterium tuberculosis* reaches the liver by hematogenous route. Hepatobiliary TB incidence is 0-1% per year. Mortality is fairly high even in cases diagnosed early (14%). Mortality is increased in patients with jaundice (62–75%) [1].

## 2. Case Report

**Figure 1:**
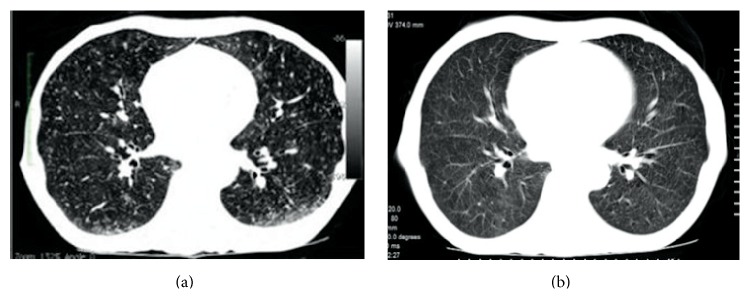
Chest CT images at (a) presentation and (b) fifth month of anti-TB treatment.

**Figure 2:**
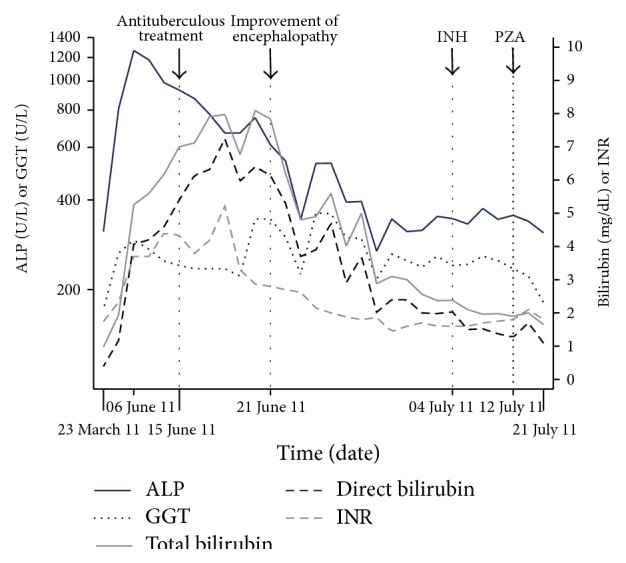
Liver function tests during the patient management. ALP denotes serum alkaline phosphatase; GGT, gamma glutamyl transferase; INH, isoniazid; PZA, pyrazinamide.

In April 2011, a 63-year-old man presented to the emergency room with chest pain and collapsed with cardiac arrest. Spontaneous circulation was achieved following cardiopulmonary resuscitation. He was referred to the hematology clinic for the evaluation of erythrocytosis before elective coronary angiography. A deep vein thrombosis was found on his right leg. He was diagnosed with polycythemia vera. Anticoagulation with enoxaparin followed by phlebotomy and acetylsalicylic acid was advised. In June 2011, he was represented with severe cachexia, jaundice, slurring speech, and grade II hepatic encephalopathy. He had a six-month history of weight loss, malaise, and night sweats and a past history of pulmonary tuberculosis (TB) 40 years ago. Physical examination revealed icterus, flapping tremor, fetor hepaticus, hepatomegaly, and splenomegaly. Prothrombin time was prolonged (34.3 seconds) and he had a low albumin level (2.62 g/dL). Serum alanine aminotransferase and aspartate aminotransferase levels were within normal ranges (32 U/L and 37 U/L, resp.). Alkaline phosphatase and total and direct bilirubin levels were high (up to 1267 U/L, 5.26 mg/dL, and 4.07 mg/dL, resp.). Supportive therapy was instituted for hepatic encephalopathy. There was no history of hepatotoxic drug intake. Viral markers including hepatitis A, B, C, and HIV were all negative. MR cholangiopancreatography revealed dilatation in intrahepatic biliary tract. Thoracic CT showed noncavitated scar in right apex with miliary seeds in the parenchyma (Figure 1(a)). Three consecutive sputum samples, gastric lavage, and urine samples were negative for acid-fast bacilli. Mycobacterial blood cultures were also negative. Diagnosis of acute liver failure (ALF) due to possible miliary TB was made. King's Collage Criteria for nonacetaminophen induced ALF indicated poor prognosis and need for immediate liver transplantation. Due to his medical comorbidities, he was considered to be ineligible for the transplantation. Rifampin (600 mg/day), ethambutol (1000 mg/day), and moxifloxacin (400 mg/day) were started. Isoniazid (INH) and pyrazinamide were spared due to ALF. Encephalopathy improved within one week of the treatment. INH (300 mg/day) and pyrazinamide (1000 mg/day) were added three weeks later when his liver functions ameliorated (Figure 2). He was discharged at the end of the eighth week of his admission. Follow-up pulmonary CT showed prominent improvement in miliary pattern after the fifth month of the anti-TB treatment (Figure 1(b)). Cultures of sputum, gastric lavage, urine, and bone marrow were negative for* Mycobacterium tuberculosis*. The treatment was given for 12 months and the treatment outcome was cure.

## 3. Discussion

Hepatic tuberculosis presents in three forms. The most common form is the diffuse hepatic involvement seen along with pulmonary or miliary tuberculosis. Despite the diffuse involvement of the liver, symptoms of liver disease are often absent. The second form is a diffuse hepatic infiltration without recognizable pulmonary involvement, also known as granulomatous hepatitis. The third, much rare form, presents as a focal/local tuberculoma or abscess. Both caseating and noncaseating granulomas are seen [2]. ALF due to miliary tuberculosis was reported in only a few cases. Miliary TB was diagnosed by postmortem examination in all those cases [3–5]. Failure to isolate* M. tuberculosis* from appropriately collected specimens in persons who are suspected of having pulmonary TB because of clinical or radiographic findings does not exclude a diagnosis of active TB. Approximately 17% of the reported new cases of pulmonary TB have negative cultures in the United States [6].

Perhaps the most important consideration in patients with TB-induced ALF would be the drug hepatotoxicity. As long as other possible causes of ALF are carefully excluded and hepatotoxic agents, such as INH and pyrazinamide, are avoided during the initial period of treatment, an almost fatal case can be treated safely.
